# Applying the Non-Adoption, Abandonment, Scale-up, Spread, and Sustainability Framework Across Implementation Stages to Identify Key Strategies to Facilitate Clinical Decision Support System Integration Within a Large Metropolitan Health Service: Interview and Focus Group Study

**DOI:** 10.2196/60402

**Published:** 2024-10-17

**Authors:** Manasha Fernando, Bridget Abell, Steven M McPhail, Zephanie Tyack, Amina Tariq, Sundresan Naicker

**Affiliations:** 1 Australian Centre for Health Services Innovation and Centre for Healthcare Transformation School of Public Health and Social Work, Faculty of Health Queensland University of Technology Brisbane Australia; 2 Digital Health and Informatics Directorate Metro South Health Brisbane Australia

**Keywords:** medical informatics, adoption and implementation, behavior, health systems

## Abstract

**Background:**

Computerized clinical decision support systems (CDSSs) enhance patient care through real-time, evidence-based guidance for health care professionals. Despite this, the effective implementation of these systems for health services presents multifaceted challenges, leading to inappropriate use and abandonment over the course of time. Using the Non-Adoption, Abandonment, Scale-Up, Spread, and Sustainability (NASSS) framework, this qualitative study examined CDSS adoption in a metropolitan health service, identifying determinants across implementation stages to optimize CDSS integration into health care practice.

**Objective:**

This study aims to identify the theory-informed (NASSS) determinants, which included multiple CDSS interventions across a 2-year period, both at the health-service level and at the individual hospital setting, that either facilitate or hinder the application of CDSSs within a metropolitan health service. In addition, this study aimed to map these determinants onto specific stages of the implementation process, thereby developing a system-level understanding of CDSS application across implementation stages.

**Methods:**

Participants involved in various stages of the implementation process were recruited (N=30). Participants took part in interviews and focus groups. We used a hybrid inductive-deductive qualitative content analysis and a framework mapping approach to categorize findings into barriers, enablers, or neutral determinants aligned to NASSS framework domains. These determinants were also mapped to implementation stages using the *Active Implementation Framework stages* approach.

**Results:**

Participants comprised clinical adopters (14/30, 47%), organizational champions (5/30, 16%), and those with roles in organizational clinical informatics (5/30, 16%). Most determinants were mapped to the organization level, technology, and adopter subdomains. However, the study findings also demonstrated a relative lack of long-term implementation planning. Consequently, determinants were not uniformly distributed across the stages of implementation, with 61.1% (77/126) identified in the exploration stage, 30.9% (39/126) in the full implementation stage, and 4.7% (6/126) in the installation stages. Stakeholders engaged in more preimplementation and full-scale implementation activities, with fewer cycles of monitoring and iteration activities identified.

**Conclusions:**

These findings addressed a substantial knowledge gap in the literature using systems thinking principles to identify the interdependent dynamics of CDSS implementation. A lack of sustained implementation strategies (ie, training and longer-term, adopter-level championing) weakened the sociotechnical network between developers and adopters, leading to communication barriers. More rigorous implementation planning, encompassing all 4 implementation stages, may, in a way, help in addressing the barriers identified and enhancing enablers.

## Introduction

### Background

The integration of digital technologies in health care services, especially the implementation of computerized clinical decision support systems (CDSSs), promises to enhance the quality, safety, and efficiency of patient care [1]. Evidence continues to build in favor of implementing CDSS for the optimization of clinical management decisions [2], thereby enabling more effective risk-based decision-making and the delivery of personalized care within the realm of acute health care [3]. Noteworthy applications include the adoption of computerized provider order entry systems [4], the deployment of point-of-care alerts to enhance patient safety [5], and the integration of electronic health record data and artificial intelligence (AI) for decision support [6,7].

Indeed, CDSSs are increasingly incorporating AI and machine learning to realize several critical benefits to clinical decision-making [8]. AI and machine learning techniques facilitate rapid analysis of extensive clinical data, including patient records and medical literature, surpassing traditional rule-based systems in swiftly generating insights and recommendations [1,8]. These technologies excel in identifying intricate patterns and relationships within data, enhancing diagnostic accuracy and treatment recommendations [1]. The relevance of AI and machine learning to CDSS adopters, such as health care providers, lies in the ability to tailor recommendations and predictions to specific scenarios [9]. For instance, machine learning can enhance diagnostic accuracy for common medical conditions by learning from large datasets, which directly benefits the adopters by providing more precise and actionable insights [10]. Although AI-enhanced CDSSs promise more precise, timely, and personalized clinical support, they are designed to complement rather than replace human judgment, necessitating careful consideration of implementation risks and limitations to optimize patient care and outcomes [11].

Despite these considerable potential benefits, the effective implementation of these systems within health services presents multifaceted challenges [1,12]. These include managing diverse stakeholder expectations, emergent clinical and sociopolitical contexts, and changing strategic priorities [13,14]. Failing to address these challenges may give rise to unanticipated outcomes related to low adoption rates [15,16], inappropriate use [17,18], unforeseen consequences [19,20], and long-term technology abandonment [21].

CDSSs can be valuable tools in health care, offering guidance to professionals; however, these systems can face limitations in practice. For example, CDSSs may struggle to accommodate patients with complex comorbidities, potentially leading to treatment recommendations that inadvertently worsen certain conditions such as prescribing heart disease medication that could harm kidneys [1]. Furthermore, CDSSs often do not incorporate patient preferences, cultural beliefs, or financial constraints as these data sources are not considered, necessitating personalized adjustments by clinicians to ensure treatment adherence and efficacy [1]. In acute care settings, CDSS may not keep pace with rapidly evolving clinical conditions, requiring clinicians to rely on their real-time assessments rather than potentially outdated CDSS guidance [1,9]. Moreover, there are high costs associated with the implementation, adoption, and maintenance of CDSSs [22,23]. These limitations of CDSSs underscore the critical role of effective implementation so that the right CDSS can provide the right information at the right time to the right patient [1].

Examining the factors that drive the successful adoption of CDSSs and those that impede its progress contributes to a deeper understanding of the dynamic interplay between technology and health care delivery [24-26]. This can be approached systematically [1,12-21] with guidance from well-established theories within the discipline of implementation science, which also accounts for organizational complexity [27,28]. A recent scoping review found that models, rather than theories or frameworks (18/42, 43% of the included studies), were most frequently used to guide CDSS adoption and evaluation strategies [29]. Unlike frameworks, models can be limited in examining the complexity of sustained implementation, acceptability, and adoption of technology across organizational and system levels [29,30]. The Non-Adoption, Abandonment, Scale-Up, Spread, and Sustainability (NASSS) framework uses a complex systems approach, which encapsulates the determinants NASSS of technological adoption overtime in health care settings [24]. This framework serves as a conceptual lens through which technological interventions are viewed as part of a complex system consisting of many processes and components. The utility of the NASSS framework lies in its ability to identify contextually appropriate determinants and inform implementation strategies, thereby shedding light on the factors that impact the success of digital health implementations [31-33]. Contextually informed implementation strategies tailored to influence clinician behavior could have a greater influence on CDSS adoption than technological design and content features [15,24-26,34].

### Objectives

As such, this research was characterized by 2 principal aims. First, we aimed to identify the NASSS theory-informed determinants that either facilitate or hinder the application of CDSSs within a metropolitan health service. These include multiple CDSS interventions at the health-service level and at a single hospital setting spanning for 2 years. Second, this study aimed to map these determinants into specific stages of the implementation process, thereby developing a systems-level understanding of CDSS application across implementation stages. A stage-specific mapping approach allows for more nuanced and tailored strategies for CDSS integration, ensuring that the unique challenges and opportunities associated with each implementation stage are addressed [35-37].

## Methods

### Ethical Considerations

The Metro South Health Human Research Ethics Committee granted ethical clearance for this research (HREC/2020/QMS/64807). All participants provided written and verbal informed consent before participating in the study. Participation was voluntary and participants could withdraw at any time. All data were deidentified and handled in accordance with the Metro South Human Research Ethics Committee guidelines.

### Study Design and Theoretical Framework

This qualitative study used a hybrid inductive-deductive approach [38]. The deductive approach was informed by the NASSS framework to identify contextually specific determinants associated with the use of CDSS technology in a large metropolitan health service across discrete implementation cycles during a 2-year period. The NASSS framework positions technological interventions as part of a complex system and has been used to guide implementation efforts and identify factors that influence technology implementation success in health services [24,31-33].

### Study Setting

This study was conducted in a metropolitan health service comprising 5 hospitals, which serve a large catchment area (3856 km^2^) in Australia. Consequently, those employed by the health service within the digital health and informatics portfolio were recruited to take part in the study. It must be noted that this department operates at both a health-service level and a facility level. In addition, clinicians who worked at either of the 2 largest hospitals within the health service were also recruited. With a facility of 1033 beds, hospital 1 is the largest teaching and training university hospital and is equipped with all major medical specialties except maternity services and pediatrics. Hospital 2 comprises most medical specialties, with 459 beds, including maternity services and pediatrics. Both hospitals (and the health service at large) used the same integrated electronic medical record system that had been implemented before the commencement of the study.

### Participant Recruitment and Sample

The participants sampled in this study included staff from the clinical informatics unit, which operated at the health-service level. In addition, clinical staff who worked within the 2 largest (defined according to the number of available beds) hospitals within this health service were also recruited to participate in the study. In this study, CDSSs were defined as any electronic system or interface designed to provide health system users with tailored information to inform decision-making within a particular context or situation [1,31]. The participants who met the following selection criteria were recruited for this study: experience with decision-making, governance, purchasing, design, and implementation of CDSS initiatives within the health service. This could include those with roles in informatics, governance, and management, as well as frontline clinical staff. The researchers used purposive sampling throughout the study, seeking representativeness of participants who were involved in using or implementing CDSS. The researchers also sought representativeness across a range of implementer roles, that is involved in making decisions or engaging in CDSS procurement, rollout, and upgrades, and adopter roles, that is clinician users of the CDSS [39,40]. To obtain this representativeness, the researchers estimated a sample size of 30 to 40 participants.

Acting as a knowledge broker, SM, an academician who is also embedded in the health service, used a knowledge brokering process [41] to identify potential participants during informal discussions with health-service staff. Those identified were then formally invited to the study using internal memos and emails sent from SM, with participants given a week to respond. Nonresponders were followed up one more time within a fortnight of the initial email. Saturation was deemed to be achieved when no new concepts or understanding were identified after 3 consecutive interviews following purposive sampling [39].

### Study Materials and Data Collection

The reporting of findings was guided by the Standards for Reporting Qualitative Research checklist (Multimedia Appendix 1). A semistructured, NASSS framework–informed interview guide, which focused on the availability, development, and perceptions of CDSS within the participants’ health system (Multimedia Appendix 2), was developed. Questions also explored decisions around the implementation of CDSS as well as their adoption, use, and sustainment in the course of time.

Data collection was conducted between March 2021 and March 2023. On the basis of the participants’ preferences and availability, data were collected through one-on-one interviews or through focus group discussions among groups consisting of 6 participants. This was done through in-person and web-based (Teams; Microsoft Corporation) techniques. Interviews and focus group discussions were led by SM, an experienced digital health and health services researcher (male, PhD qualified, embedded in health service, and familiar to a few participants), and SN, an experienced mixed methods health services researcher (male and PhD qualified). The interview duration was approximately 1 hour, while focus group discussions were conducted for up to 2 hours. The interviews and focus group discussions were audio recorded and transcribed verbatim. Preliminary findings were monitored and discussed with the research team. This information was used to guide recruitment until saturation was reached [33,42].

### Obtaining Saturation

Initially, SN analyzed the transcripts thematically and concurrently with data collection to facilitate purposive sampling and identify saturation. Early analysis of transcripts allowed us to identify preliminary themes and gaps, informing subsequent interviews and ensuring that a diverse range of perspectives were explored [33,42,43]. This iterative process of concurrent analysis and interviewing was integral to refining our understanding and ensuring the trustworthiness of the findings [42,44]. At the completion of the interviews and focus group discussions, MF and SN analyzed data using qualitative content analysis [31] and framework analysis [31,45-47]. Framework analysis is a systematic qualitative analysis widely used in health and social care research to organize and analyze large volumes of textual data, enabling comparison by case and theme [48].

**Figure 1:**
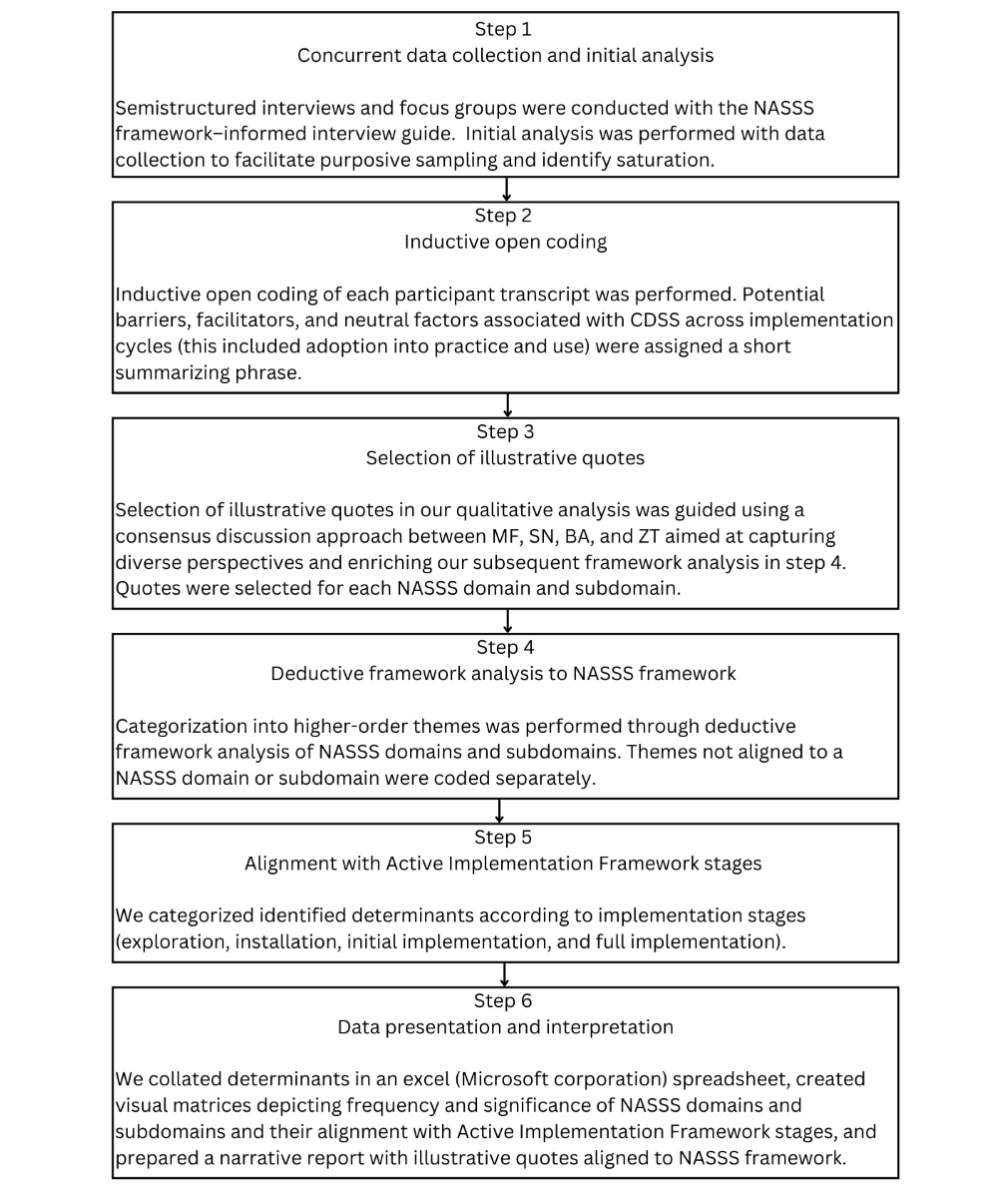
Analytic process used in data analysis. CDSS: clinical decision support system; NASSS: Non-Adoption, Abandonment, Scale-Up, Spread, and Sustainability.

The data analysis process is outlined in Figure 1. First, barriers, facilitators, and neutral factors associated with CDSS across implementation cycles were assigned a short summarizing phrase [31,42]. This was done through inductive open coding of each transcript. These initial phrases were discussed and revised, with a few being discarded, amended, or subsumed to create higher-order codes [31]. Through an initial inductive analysis of codes, the researchers aimed to capture the context-specific elements of the data without prematurely applying a predefined framework [38,46]. This approach enabled the discovery of preliminary themes and patterns, which were subsequently aligned with the NASSS domains and subdomains during the deductive phase of the analysis [33,38,46].

### Alignment With the NASSS and Active Implementation Stages Frameworks

As a part of this process, where appropriate, illustrative quotes were extracted for each of these codes [42]. The selection of illustrative quotes during qualitative analysis was guided using a consensus approach after discussion with MF, SN, BA, and ZT. The selection of quotes aimed to capture diverse perspectives and enrich subsequent mapping to the NASSS framework to showcase the variations within each theme and subtheme, ensuring that findings were both grounded in the data collected and demonstrative of the range of perspectives expressed by the participants [33,45,46,48-50]. The outcome of this process was to reflexively map each of the higher-order themes onto ≥1 of the NASSS domains and subdomains with which they were aligned [31]. This analysis was primarily deductive, with the intent to align barriers, facilitators, and neutral factors identified in our inductive analysis with preexisting domains within the NASSS framework [31]. It is important to note that, in the analysis, no restriction was placed on the number of domains or subdomains with which an individual-coded barrier, facilitator, or neutral factor could align [31]. This was not only to categorize using the NASSS framework but to reflect on the role of the theoretical framework in capturing the complexity of CDSS implementation within a real-world health service [31,51]. Themes not aligned to an NASSS domain or subdomain were coded separately [46]. Finally, the Active Implementation Framework [35,36] was used to categorize and display identified determinants according to the stages for digital health implementation. These stages include exploration, which is focused on the feasibility and organizational readiness, installation, which is centered on the organizational preparation, initial implementation, which covers implementation-initiation techniques such as training, and full implementation, which emphasizes sustainment [35-37].

### Data Presentation and Interpretation

A descriptive numerical summary of the identified determinants was collated in an Excel (Microsoft Corporation) spreadsheet, mapping determinants to the NASSS framework domains and the Active Implementation Framework stages [31,42]. To illustrate the alignment of barriers, facilitators, and neutral factors with the NASSS framework and the Active Implementation Framework, the researchers developed visual matrices to depict the frequency and salience of the identified themes and their association with various implementation phases [31]. This visual and numerical representation aimed to demonstrate common findings across the NASSS domains relating to CDSS implementation and to identify gaps in the implementation of CDSS across different phases.

### Trustworthiness

Throughout the analysis, the researchers maintained trustworthiness and reflexivity by reflecting on the research process through discussion as a research team and considering how their perspectives and the chosen frameworks may have influenced data interpretation [45-47,49]. This study provided a narrative report of the framework mapping to each NASSS domain and subdomain supported by direct quotes from the interviews and focus groups [33,45,46] and discussed the implications for practice.

## Results

### Participant Characteristics and Implementation Roles

A total of 30 participants, including implementers, decision makers, and CDSS end users, across several departments took part in this study. Table 1 provides comprehensive demographic information about the participants.

**Table 1:** Participant demographics (N=30).

Position and categories		Participants, n (%)
**Sex**		
	Female	13 (43)
	Male	17 (57)
**Portfolio^a^**		
	Medical specialty (ie, radiology, pharmacy, and cardiology)	16 (53)
	Digital health and informatics directorate	8 (27)
	Nursing and allied health	6 (20)
**Job role**		
	Physician (ie, residents, junior physicians, consultants, specialists, and directors)	18 (60)
	Clinical informatics (ie, project manager, senior management, and data analyst)	6 (20)
	Allied health and nursing (ie, physiotherapist and triage nurse)	6 (20)
**Implementation role**		
	Adopter-clinician (ie, physicians, allied health staff, and nursing staff)	14 (47)
	Organizational staff–implementer informatics	5 (17)
	Adopter-clinician and organizational staff champion	5 (17)
	Adopter-clinician and organizational staff leadership	4 (13)
	Adopter-clinician and organizational staff-implementer informatics	1 (3)
	Wider system–interorganizational networker	1 (3)

^a^Portfolio refers to the health service department associated with the participant, recognizing that some participants had a clinical background, that is, physician, but were experienced at a department level with decision-making, governance, purchasing, design, and/or implementation of clinical decision support system initiatives within this health service.

**Figure 2:**
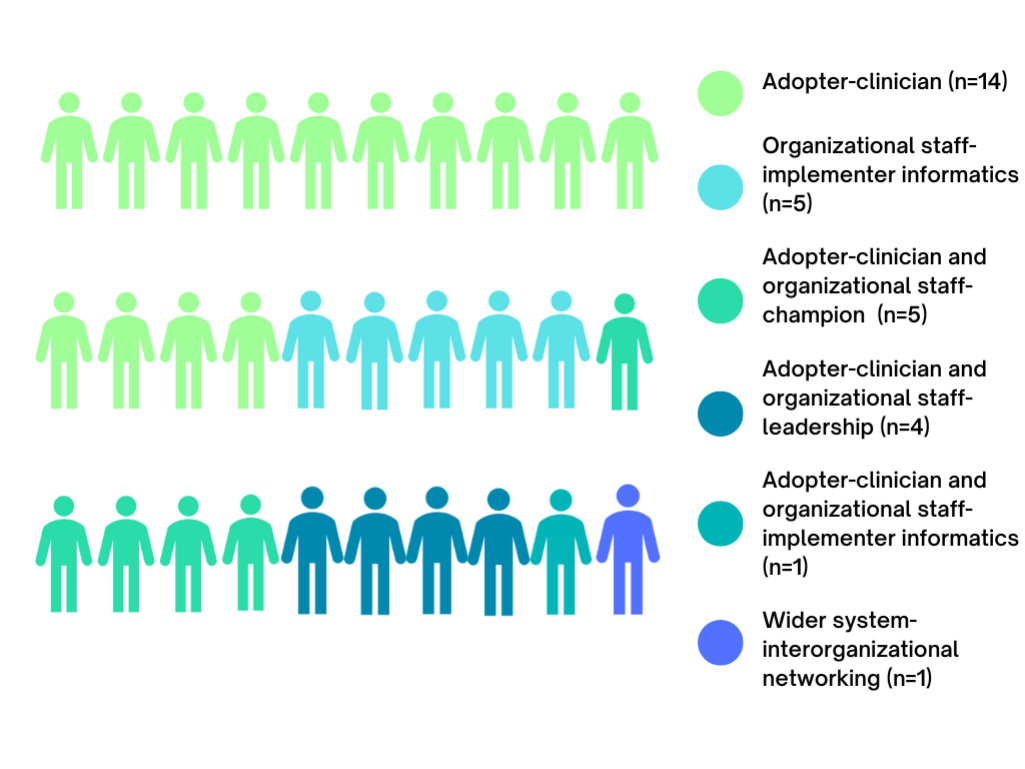
Non-Adoption, Abandonment, Scale-Up, Spread, and Sustainability (NASSS) framework–informed implementation roles of participants (N=30).

Participants were mapped to NASSS-informed implementation roles, highlighting their level of decision-making within the hospital systems of interest, as shown in Figure 2. Of the 30 participants, 14 (47%) clinical staff identified solely as adopters of CDSS tools. In addition, 17% (5/30) of clinical staff took on the role of organizational staff champions, that is, clinicians who advocate for the technology and its use [52,53]. Overall, 17% (5/30) of participants were informatics professionals, 13% (4/30) of participants acted in dual roles of organizational staff leadership and clinical adopters, that is, as clinical directors. Only 1 (3%) participant encompassed adopter-clinician and organizational staff–implementer informatics roles, that is, as a clinician who worked within the digital health and informatics directorate portfolio. Only 1 (3%) participant was considered a wider system–interorganizational networker.

### Key Determinants Associated with CDSS Implementation

**Figure 3:**
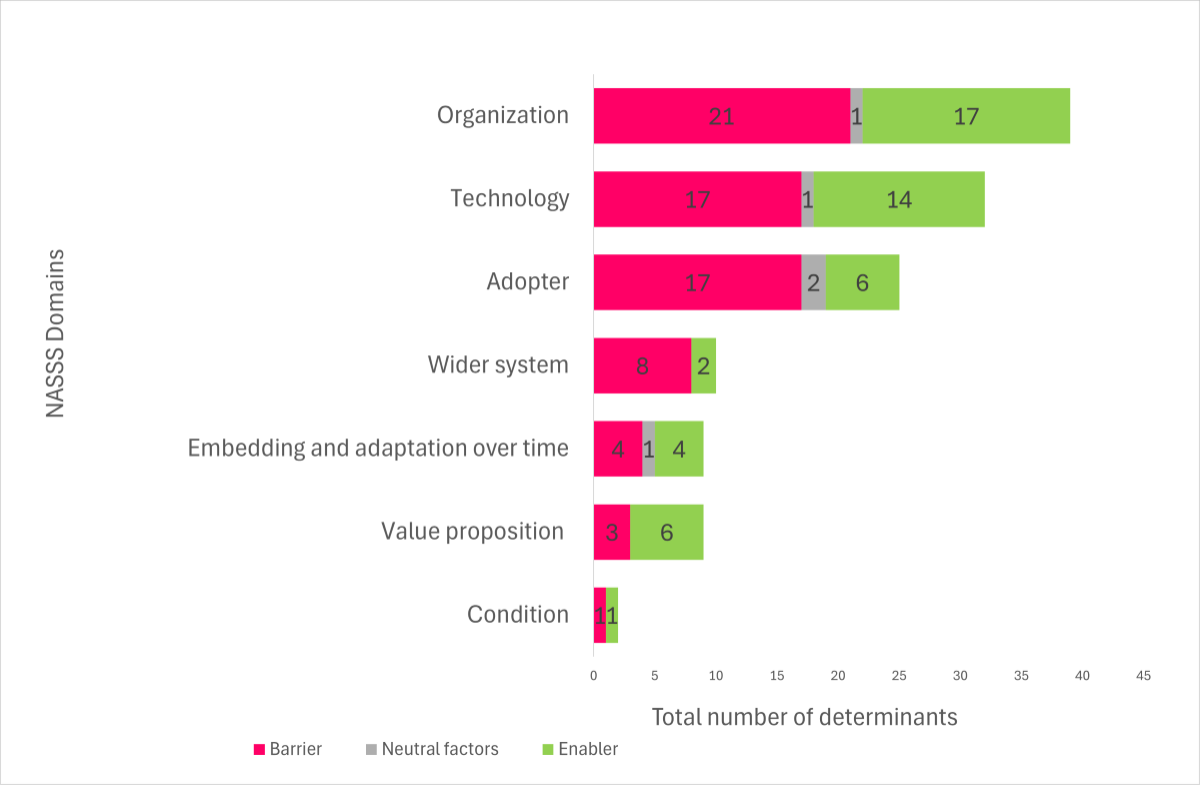
Key determinants associated with clinical decision support system (CDSS) implementation mapped to the Non-Adoption, Abandonment, Scale-Up, Spread, and Sustainability (NASSS) domains.

The identified determinants encompass specific elements pertinent to CDSS implementation. These elements were classified into barriers, enablers, and neutral factors. For a comprehensive breakdown of these determinants, refer to Multimedia Appendix 3. The findings were mapped to the NASSS domains and subdomains, as shown in Figure 3. Barriers (71/126, 56.3%), enablers (50/126, 39.6%), and neutral factors (5/126, 3.9%) associated with CDSS implementation were identified in this study. Most determinants were mapped to the organization (39/126, 30.9%), technology (32/126, 25.3%), and adopter (25/126, 19.8%) domains.

### Framework Analysis of NASSS Domains and Subdomains

#### Organizational Domain

Key organizational level determinants were mapped to the following NASSS subdomains. The barriers included limited implementation capacity stemming from a lack of longer-term implementation planning know-how:

That’s something that’s a little bit vague for me, do we plough on? With a state that shows it works. Then you bring in your usability. Or do you bring in your app and then do your study?...And so that, that design of the next phase sort of up here, but I don’t know the best way to do it would be...how I’d explain it.

Clinical adopter, senior management

The participant highlights a concern on whether to continue with existing approaches that show some amount of success (eg, *state that shows it works*) or to introduce new technologies (eg, apps) and evaluate their impact. This decision-making process ties into limited implementation capacity, underscoring the importance of strategic planning and expertise in guiding successful implementation efforts. This may have flow-on effects, resulting in inappropriate resource prioritization, which is identified as a barrier that needs to be worked on:

Generally, they will provide support for when they’re rolling out something new. But often you don’t actually discover issues or problems with that new process or software until you’re using it.

Clinical adopter

Furthermore, this impacted *technological readiness* as a barrier, as stated by a participant:

I think [Health Service] really hasn’t accommodated us because...if you’re on a VPN connection, you’re throttled down to three or four megabits per second.

Clinical adopter

While not every clinician in the hospital may always be on a virtual private network (VPN), VPN throttling affects system performance in hospitals by limiting bandwidth, thereby hindering the speed and efficiency of accessing critical systems or data. This can negatively impact workflow efficiency and user satisfaction for those required to use a VPN. This quote also reflects participant’s concerns about organizational readiness to adopt new technologies effectively in comparison to existing organization infrastructure and practices. Conversely, the organization appeared well- equipped to support initial or early-stage CDSS implementation activities, including training:

Champions are trained, they’ve done validation, then it’s a case of, OK, now we go live with that product.

Informatics staff, senior management

The organization has also established a highly skilled clinical informatics workforce, as stated by a participant:

They’re the only group that has a clinical informatics solution. So, you can do a training...in clinical informatics. So, it’s nice because they have some champions now integrated.

Clinical adopter, digital consultant

This workforce ensures ongoing maintenance and support in navigating the digital ecosystem:

There is a department in place to help you navigate all the things you need to do. Whether that’s from an IT procurement perspective, whether or not that’s from a cybersecurity perspective...

Informatics staff

These enablers were mapped to the subdomain of work needed to plan, implement and monitor change. It must also be noted that the inclusion of organizational champions across clinical contexts was a significant enabler in the adoption of several CDSSs across this hospital system. This was a key factor in enabling the initial work needed to plan, implement, and monitor change.

The informatics team has a close familial culture due to the team members working together for a long time:

All of us have been working together for 10 plus years, some of them 15. Very close-knit team. So that is what holds us together.

Informatics staff

The tight-knit nature of the informatics team can be seen as part of the organizational culture, impacting how the team works together, their ability to innovate, and their readiness to implement changes in the organization. Although mapped to the organizational domain of the NASSS framework, given that the primary focus of this culture was not strictly covered by standard organizational structures or leadership theories associated with the NASSS framework’s *general capacity to innovate* [54] but instead highlighted the unique interpersonal dynamics within the team, we coded this separately from the NASSS framework.

#### Technology Domain

In the hospital system, the most common technological barrier was associated with the interoperability of CDSSs, which was mapped to the material properties subdomain:

Sometimes, the lack of interface between the systems...they run a parallel system.

Clinical adopter

Participants also shared that they were not always fully aware of CDSS modifications:

There used to be a feature in the IEMR where you could taper medication doses...I don’t know what ever happened to it and it just wasn’t there anymore.

Clinical adopter

In addition, users were not always aware of the best ways to optimize using CDSS technology in this hospital system:

I know as someone who works in digital health and informatics, there is...a little button to the side of that that says, Click to see the ‘information. But I don’t think a lot of people realize that...

Clinical adopter, digital consultant

However, when participants were able to experience how CDSS supported effective practice, they were more likely to engage and use the technology:

If you’re not too familiar with this medication or you’ve forgot what the maximum dose should be or you were distracted by something else, the power plan [CDSS] has it there and says you shouldn’t be going above this...so I think that’s good from a patient safety perspective.

Clinical adopter

I would imagine ease of use for a start...if it’s too clunky, they won’t go anywhere, you know, even introducing a new app into the system if it...takes 10 minutes to set it up.

Clinical adopter, senior management

Ease of use drives clinicians’ confidence.

Clinical adopter, senior management

These quotes are illustrative of how *ease of use* can enhance clinicians’ overall confidence and proficiency in using the technology effectively. This aspect minimizes the learning curve and allows users to operate the CDSS with minimal training or interruption. This can lead to increased confidence among clinicians because they are able to interact with the system more efficiently and focus more on patient care rather than struggling with the technology. Therefore, this enabler mapped to the NASSS subdomains of knowledge generated by the technology and knowledge to use it.

#### Adopter and Condition

Despite some participants viewing technology as supportive of effective practice, others expressed concerns with machine learning found in modern CDSSs. These concerns were about limiting the capacity for critical thinking, professional autonomy, and personal legal ramifications:

The threat of machine learning would be that you could become reliant on, you know, what an algorithm is telling you and directing you to do, and you might lose that art of being able to, like, go well...I recognise this issue because I’ve seen it before.

Clinical adopter

This is where I think it’s something we’re going to have to have to work out and develop some protections around it...I want to make sure this thing is safe.

Clinical adopter

Conversely, adopters expressed positive perceptions of CDSS technology when there was a clear lived experience of improved workflow:

From a workflow perspective, it reduces the number of clicks and the number of individual actions you need to do to...So I think it makes things a lot easier in that respect.

Clinical adopter

This was also the case for patient-centered care observed through improved outcomes:

I think that’s...the excitement of machine learning is learning more...individualising the dose for a patient.

Clinical adopter

Effective adaptation to the local clinical context was also an enabler, as stated by a participant:

One sort of springs to mind that we do use as a screening tool...It’s helpful to do it, sort of pops up for every patient during your initial clinical assessment...it’s just like three little questions that you ask...it is an incentive for me to do a complete set of vitals each time...I feel like I do it more often because I’ve got that incentive to get that score.

Clinical adopter

Participants emphasized the importance of adapting CDSSs to local clinical environments. CDSS was viewed positively when demonstratively relevant adaptations were made in contrast to a clear lack of clinically contextual adaptation:

Sometimes it can be a bit too rigid, and if you’re wanting to do something that falls out of the power plan, they sometimes can make the job harder...

Clinical adopter

The warfarin one works well and I think it's a better example of how our plans would be used.

Clinical adopter

Consequently, this determinant was also mapped to the condition domain.

#### Value Proposition

CDSS using AI was seen as valuable to adopters by enabling efficiency in protocolized tasks with predefined workflows:

So, it’s really saving time on things that can very well be done by sort of an artificial intelligence...it won’t replace the reporting.

Clinical adopter, senior management

In contrast, when considering the relationship between demand-side value (to adopters) and supply-side value (to developers), different priorities can create misalignment regarding the value proposition of a CDSS initiative. This misalignment may lead to communication barriers:

I think our clients and customers, they think they’re things really important. Because they have no visibility of the strokes that we’re managing at the moment, yeah so. For them, it’s hard to say what do you mean. My initiative in one tiny little ward, which means so much to me...you can’t help me. And I think, because truthfully, they don’t have visibility of all the other things that we’re doing.

Informatics staff

#### Wider System

The lack of clear and consistent governance surrounding the application of CDSSs within the hospital context was perceived as a major wider system barrier by most participants:

But it’s also really raised a lot or highlighted a lot of the areas where there could be more maturity...I guess, operating in terms of making sure that whatever processes need to be in place for governance and decision-making, ethics, liability.

Informatics staff, senior management

However, participants also noted that the experience of the COVID-19 pandemic showed that wider system decision-making to facilitate the appropriate use of digital technology could be streamlined in an effective and efficient manner:

I think COVID certainly showed us that we can streamline a lot of our decision making...

Clinical adopter, digital consultant

#### Embedding and Adaption With Time

Participants noted the resilience of this hospital system in adapting to and accommodating the application of technology as a key strength. This stemmed from an innovation culture and recognition of the importance of evaluating technology across implementation cycles, even if they did not currently have the resources or exact know-how to do so:

I’m a massive champion of that whole point of we do go through a cycle, right? Where you plan your budget, you deliver, you maintain most systems...go to that next step, which is to evaluate and more importantly, evaluate does this thing still deliver the same value statement that we thought it would at the start.

Informatics staff

A noted barrier was the acknowledgment that adapting complex CDSSs, particularly AI, to contextual changes in the system in the course of time was specialized and labor intensive:

So, we need to optimise it...how do we then make sure that the tool stays accurate over time? No one really has, I think, quite worked out, I think “that’s going to be the challenge.”

Clinical adopter, senior management

### Mapping NASSS-Informed Determinants to Implementation Stages Across the Health Service

#### Overview

**Figure 4:**
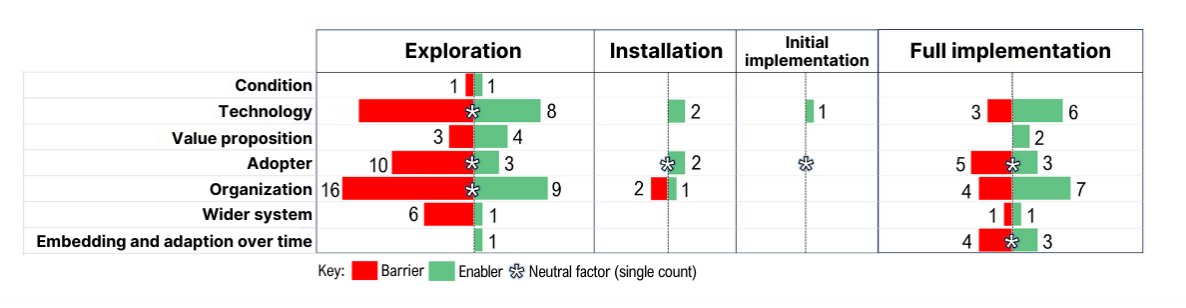
A tabular diagram illustrating the Non-Adoption, Abandonment, Scale-Up, Spread, and Sustainability (NASSS) determinants mapped to implementation stages.

It must be noted that when mapped to Active Implementation Framework stages [35-37], there was an uneven distribution of determinants across implementation stages, with most determinants falling in the scope of the exploration and full implementation stages. This highlighted a tendency of stakeholders to engage in the frequent preimplementation or exploration activity and full-scale adoption activity. In contrast, fewer determinants were associated with identifying contextual drivers, developing adopter readiness, and facilitating contextual capacity building to sustain ongoing adaption within an established feedback and monitoring strategy. Figure 4 illustrates the uneven distribution of the 126 identified determinants; Out of the identified determinants, 77 (61%) were solely associated with the *exploration* stage, 39 (31%) with the *full implementation* stage, and 6 (5%) with the *middle installation* stage.

#### Exploration

Most determinants described in this study were identified during the preimplementation stage, which is associated with assessing organizational readiness and technological procurement (exploration stage). Enablers in this stage included mapping CDSS procurement to the clinical context and recognizing the importance of involving diverse stakeholders. Conversely, there were several barriers relating to the organizational, technological, and wider system within the exploration stage. This included a lack of streamlined executive decision-making for CDSS procurement and adaption and navigating complex governance processes.

#### Installation and Initial Implementation

These stages describe actions associated with identifying contextual factors, which may facilitate the integration of a CDSS into a system to inform an initial smaller-scale rollout or pilot. The organization appeared to have engaged in fewer installation-stage piloting activities; however, the key enabling determinants were identified. Among these adopter-level factors associated with enhancing clinician trust in CDSSs through education, training, and leveraging peer champions were noted. Conversely, barriers in this stage included a lack of targeted resourcing to facilitate full-cycle CDSS implementation planning.

#### Full Implementation

This stage represents a state of full technological integration across an entire system or organization. This involves activities associated with ongoing monitoring and iteration, scalability, and replicability. Several enablers were identified within this phase, particularly at an organizational and technological level, when considered in the context of adaption and embedment in the course of time. This included decision makers recognizing the need for ongoing investment in capacity building to sustain a digitally informed workforce and the need for ongoing iteration of CDSSs to support effective practice in the course of time. The barriers identified included the lack of sustained organizational champions postadoption and insufficient training and information on technology upgrades which occurred over time.

#### Multistage Implementation Activities

Four determinants spanned multiple stages. Organizational-level training of champions supported CDSS adoption in both exploration and installation stages. A notable barrier was the lack of knowledge and guidance for implementation planning, affecting organizational readiness. Trust in CDSS was recognized as varying among clinicians and was not systemically evaluated across all implementation stages. Inbuilt tracking mechanisms to measure uptake and patterns of CDSS were seen as beneficial for transparency and user fidelity throughout implementation cycles but were not uniformly explored or applied.

## Discussion

### Principal Findings

By mapping NASSS-informed determinants influencing CDSS implementation cycles using the Active Implementation Framework stages [35-37], our research provides pragmatic insights to inform tailored integration strategies of these technologies into large hospital systems. Despite encountering limitations in implementation capacity and planning know-how, the institution demonstrated strength through a well-equipped clinical informatics workforce and early-stage training of its adopter workforce. This finding aligns with existing literature, emphasizing the critical role of organizational culture and support structures in successful technology adoption [13,31]. Second, CDSS implementation faced technological hurdles, including interoperability and interface issues, a frequently reported CDSS engagement barrier [15,22,55]. However, positive user experiences emerged as a significant factor influencing CDSS use, particularly when adopters could directly experience improved efficiency and better patient outcomes for themselves. This underscores the importance of user-centric design and showcases the practical impact of CDSS on health care workflows, aligning with the broader pattern of emphasizing end user perspectives in technology adoption [12,15,17]. The presence of peer champions among clinicians emerged as a significant enabler. Peer-to-peer support and advocacy played a crucial role in enhancing clinician confidence and acceptance of CDSSs, contributing to a smoother initial implementation process. Moreover, the study reveals wider systemic barriers, such as a lack of clear governance for CDSS applications, aligning with the ongoing discourse on the necessity for robust regulatory frameworks in the broader implementation of digital health care technologies [25,56]. This finding emphasizes the need for a systemic approach to address governance gaps and ensure the effective integration of CDSSs into health care systems. These findings strongly align with our recent scoping review, which mapped reported CDSS implementation barriers and enablers to NASSS domains [31]. Although further empirical evidence from successful implementation is required, this further highlights the reliability of the NASSS framework as a pragmatic tool to identify meaningful domain-level determinants associated with the implementation of technology within health systems [24,54].

When determinants were mapped to the *Active Implementation Framework stages* [35-37], a clear pattern emerged highlighting implementation activities most notable in the early stage of *exploration* and the later stage of *full implementation*. The absence of planned piloting and process evaluation was not unexpected, given a lack of evaluation and implementation planning *know-how* was identified as a key organizational readiness barrier.

This may be addressed through the application of theory-informed approaches to implementation planning [27,30]. This finding aligns with the gaps identified in our recent scoping review examining the use of theories, models, and frameworks to support CDSS implementation within hospital systems [29]. This review [29] reported an inconsistent application of systematic approaches to implementation planning within hospital systems in several countries.

Furthermore, while organizational-level training of champions was crucial and supported CDSS adoption in both the exploration and installation stages, this was not sustained throughout the implementation life cycle. This weakening of the informatics-adopter sociotechnical network during the course of time may have contributed to the communication issues adopters frequently highlighted. This study identified 3 key communication issues in the implementation of CDSSs. First is the challenges in effectively communicating and coordinating within the complicated web of governance structures, potentially impeding the progression of CDSS implementation. Second, the limitations in ongoing communication about system updates and advancements may lead to adopters being unaware of improvements, impacting their ability to fully use the CDSS. Third, trust in CDSSs was recognized as varying among clinicians, indicating a potential communication challenge in conveying the benefits and reliability of the system uniformly across all CDSS adopters. More rigorous implementation planning, encompassing all 4 stages, may go some way in addressing the identified communication gaps [27,29,30,35,36].

Furthermore, this study identified the need for ongoing adopter support and planned evaluation strategies encompassing the full implementation cycle [36,56]. Evidence indicates that sustained cycles of monitoring and iteration may lead to sustained integration of CDSS in health care systems [21,34,57]. For example, planned mechanisms for user tracking allow for real-time assessment of uptake, which may impact the choice and delivery of implementation strategies to facilitate the continuous adoption of the innovation in practice [10,58].

### Strengths and Limitations

The study’s strengths lie in its holistic systems thinking approach, which provides a comprehensive understanding of the challenges and enablers within each implementation stage and across implementation cycles for complex systems such as CDSS. The researchers acknowledge that the final phase, defined as *full implementation* within the *Active Implementation Framework*, does not fully address all components of sustainability, which can include factors such as changing legislation, government (federal and state) budgets, and workforce capacity [35,36,57,59]. However, this framework does acknowledge factors that bridge the policy gap within an organization, including clear governance pathways and longer-term resourcing, in addition to recognizing embedded behavior change and ongoing monitoring and iteration as parts of full implementation cycles [35].

It must be noted that while this study was intentionally designed for a 2-year period, there may have been specific events (including the COVID-19 pandemic) that may have influenced participant perceptions. However, it was not within the study’s scope to contextualize a comprehensive list of events that could have impacted perceptions. Nonetheless, our questions were designed to probe reasons behind participant perceptions when interviewed; early analysis of transcripts allowed us to identify preliminary themes and gaps, ensuring that a diverse range of perspectives were explored and stopped when no new insights were gained [33,42,44]. The participants’ roles can influence their perceptions and experiences with CDSS implementation. Clinical adopters might focus more on usability and patient impact, whereas senior management or informatics professionals may prioritize technical challenges and integration issues [60]. Findings may not fully generalize to other health care settings with different organizational structures, levels of technological maturity, or cultural contexts [10]. It must be noted that patient perspectives were outside the study scope, which focused on clinicians as end users within an acute-care (hospital) setting. Recommendations and insights derived from this study may need to be interpreted cautiously in broader contexts and may apply more directly to the study settings where similar participant distributions and roles are prevalent [10,61]. Future work should also consider examining patient perspectives to enhance insights.

The study was conducted within a specific health care system, potentially limiting the generalizability of some findings. However, the use of the NASSS framework has identified multilevel determinants that align with trends in contemporary research findings across the health care system [31,62,63]. Moreover, the study’s participant population represents a broad spectrum of stakeholders involved in CDSS implementation, including clinical staff adopters, organizational champions, and informatics professionals. This diversity ensured the representativeness of perspectives needed to understand the broader contextual factors applicable beyond this study’s settings [63,64]. By including individuals from various departments and decision-making levels, the study captures a holistic view of the challenges and facilitators influencing CDSS adoption, contributing to a nuanced understanding of implementation dynamics within the hospital system. Furthermore, this qualitative study was exploratory in nature and enabled us to unpack the intersectionality of multiple determinants in influencing a range of implementation outcomes.

Future research could extend the use of the NASSS framework to the application of CDSS in different health care environments or medical conditions such as in rural hospitals or mental health care [31,34]. Furthermore, investigations might integrate a systems-level framework, such as the NASSS framework, using behavioral assessments, standardized psychological tests, or multimodal methodologies to explore individual emotional reactivity for CDSS adoption [13,65]. Such endeavors may deepen insights into health care professionals’ emotional responses, such as stress levels, satisfaction, and confidence [13], to CDSS use. The insights gained could contribute to broader knowledge about technology implementation across diverse health care contexts, informing tailored strategies for improved adoption, long-term viability, and best practices to enhance CDSS adoption in various health care settings [31,66]. Additional quantitative data and further empirical application of the NASSS framework may be beneficial in further exploring specific qualitative findings identified in this study. Future research could explore the longitudinal aspects of CDSS adoption to capture changes and adaptations over extended periods by conducting trials with integral process evaluations to test the identified implementation factors across diverse populations and settings [53,60,63].

### Conclusions

These findings address a significant knowledge gap in the literature using system thinking principles to identify the interdependent dynamics of CDSS implementation. In moving forward, this study serves as a catalyst for informed decision-making in CDSS implementation, offering actionable insights for practitioners and researchers alike. As technology continues to evolve and health care landscapes transform, the lessons gleaned from this study provide a foundation for refining CDSS implementation strategies and advancing patient-centered, efficient, and ethically sound health care practices.

## Appendix

Multimedia Appendix 1 Standards for Reporting Qualitative Research checklist.

Multimedia Appendix 2 Non-Adoption, Abandonment, Scale-Up, Spread, and Sustainability (NASSS) framework–informed interview guide.

Multimedia Appendix 3 Supplementary breakdown of mapping.

## Abbreviations

AI: artificial intelligence
CDSS: clinical decision support system
NASSS: Non-Adoption, Abandonment, Scale-Up, Spread, and Sustainability
VPN: virtual private network

## Data Availability

The dataset (which includes individual transcripts) is not publicly available due to confidentiality policies.
