# Supplementary material for: Applying the Non-Adoption, Abandonment, Scale-up, Spread, and Sustainability Framework Across Implementation Stages to Identify Key Strategies to Facilitate Clinical Decision Support System Integration Within a Large Metropolitan Health Service: Interview and Focus Group Study
Source: JMIR Med Inform. 2024 Oct 17;12:e60402. doi: 10.2196/60402 (PMC11528173; doi:10.2196/60402)
Supplement: Multimedia Appendix 2 [file medinform_v12i1e60402_app2.docx]

Appendix

## **Appendix B**

# **Understanding the implementation and use of digital information in [REDACTED] Hospital and Health Service**

# **INTERVIEW GUIDE**

The following guide is intended to be used to conduct interviews for the *Understanding the implementation and use of digital information in* [REDACTED] *Project.*

## **Objectives:**

## To map the current digital information landscape within [REDACTED], including engaging with key stakeholders, and conducting needs assessment

## To investigate the contextual factors that have contributed to, or impeded, successful implementation, adoption, and sustainability of key clinical informatics initiatives in [REDACTED] hospital and health service

## **Participants:**

Interviews will be held with key [REDACTED] hospital and health service stakeholders involved with design, implementation and maintenance of digital interventions. Participants will be invited by their unit director or Chief Information Officer to participate via email. Participant information sheets and consent forms will be emailed with the invitation. Purposive sampling will be used to ensure interview participants are representative of the roles and projects within the [REDACTED]hospital and health service priority areas already identified.

Participants will be required to take part in an individual interview or focus groups. These may occur face-to-face, or virtually (phone or videoconference). Individual interviews will last a maximum of 60 minutes or focus groups up to two hours.

## **How to use this guide:**

This guide has been informed by the domains of the non-adoption, abandonment, scale-up, spread, sustainability (NASSS) framework^1^ to ensure all aspects of digital health implementation are considered. It is divided into sections based on the participant’s role as a technology developer/designer, organisational lead, technology user, organisational horizon scanner and clinical specialist. The interviewer should follow the questions and prompts from the section(s) of the guide that most appropriately match the interviewees role(s). Within each section:

## All key concepts should be covered; however, it is not necessary for interviewers to ask questions in the proposed order, or ask all questions exactly as stated, or repeat a question if it has been answered in an earlier section

## The conversation should explore issues as they are raised, with guidance and prompting as required

## Facilitators will ensure discussion progresses in a timely, yet informative manner

## **References**

Greenhalgh T, Wherton J, Papoutsi C, Lynch J, Hughes G, Hinder S, et al. Beyond adoption: a new framework for theorizing and evaluating nonadoption, abandonment, and challenges to the scale-up, spread, and sustainability of health and care technologies. Journal of medical Internet research. 2017;19(11):e367

## **
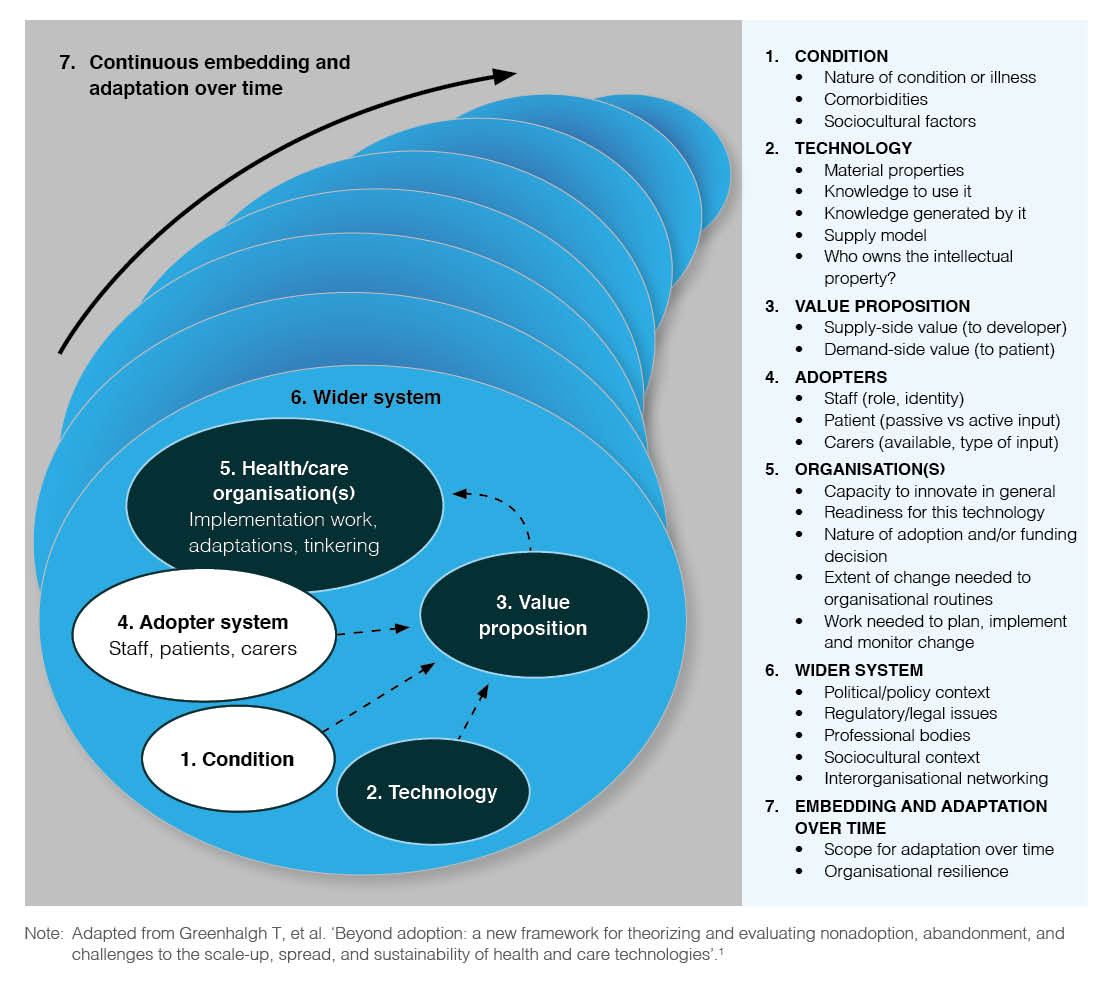
**

### Diagram: The NASSS framework (© Greenhalgh at al J Med Internet Research 2017; 19 (11): e367)

## **Interview Script**

*This section is included as an example of introductory text for the interview. Please note this can be adjusted and amended to suit the interviewer, context, format of delivery etc.*

Hi, my name is [________]. I’m speaking with you today because we are interested in understanding how digital systems and their associated information are currently being used to improve patient care within [Redacted] health service. This will inform directions for future activities and provide an evidence base to support the implementation, effectiveness, and cost-effectiveness of clinical informatics initiatives. We want to get more information about what initiatives are being implemented, how these are chosen, and the factors that influence or impede successful adoption, uptake and sustainability among staff. You have been selected as a key stakeholder, and we believe you have important information to share with us due to your role in [__insert role/team__].

We will record the conversation with you if that is OK and will only share your conversation with other members of the research team or by making sure other people cannot identify your responses. We really appreciate your time and willingness to speak with us. Do you have any questions before we get started?

## **Interview Questions**

First, we’re going to talk about some of the clinical decision support solutions that you’ve seen implemented here before, and then think more broadly about the readiness of your team and [Redacted] health service for embracing digital health technologies.

1. **Could you tell me about your current role as it relates to clinical decision support?**

*Prompts***:**

- *How do you conceptualise (think about), decision support systems in your current role?*
- *In what ways do you think it could improve healthcare?*
- *In what ways do you think it could disadvantage healthcare?*
- *How do you keep up to date with knowledge in this area?*

1. **In your experience could you tell me about some of the main computerised decision support tools that have been implemented in your unit/department/clinical area/ hospital/health service?**

*Prompts:*

- *What type of technology did it use?*
- *What processes did it impact on? E.g. triage, decision-making, risk management*
- *Where did it come from? E.g. developed in-house, outsourced or a blend of both approaches?*
- *Who was involved in making the key design/procurement decisions?*
- *How was it implemented?*
- *Have you ever been involved in the work of implementing some of these systems? If so, can you tell me about the key parts of that process?*

1. **Were there any implementation gaps with any of the systems/tools you mentioned? If so, could you tell me a little about it?**

*Prompts*

- *Were there any barriers to implementation?*
- *Were there any enablers (systems, organisational support, environmental and context) that made implementation into the system smoother?*

1. **In your experience could you tell me about your perceptions /opinion and experiences with some of these computerised clinical decision support systems you mentioned?**

*Prompts:*

- *How have they impacted your workflow?*
- *How have you used them to support your objectives/clinical practice decisions?*
- *Instances when these tools/systems have failed and how it was handled?*
- *Do have enough support and training to use these systems effectively?*

1. **What kind of feedback have you received by end users regarding how these tools have been accepted and used? (For informatics, senior management adopter staff and champions)**

*Prompts:*

- *What are the challenges of actually using it in practice?*
- *Have you observed people trying to use it? What do they say?*
- *Is it easily obtainable and dependable (e.g. doesn’t crash)?*
- *Do users understand what it does and the data it generates?*
- *Does it require major changes to organisational tasks and routines?*
- *What help is offered to users (e.g. helpdesk, hands-on support)?*
- *Are there multiple technical interdependencies or upgrades required?*
- *Are there any privacy or security concerns?*

1. **How has this impacted workflow changes, if at all?**

*Prompts:*

- *Are different kinds of staff (e.g. new hires) involved in the process or pathway once the technology has been introduced?*
- *Do new (or radically revised) processes or pathways need to be developed?*
- *Do core processes or pathways need to link differently with other key processes and pathways in the organisation?*

1. **How would you evaluate the value proposition of these tools?**

*Prompts:*

- *Has the technology been shown to have an overall advantage over existing practice?*
- *Has technology been shown to be effective and cost-effective?*
- *Is the technology and its impact desirable for patients?*
- *Are there concerns that the technology, whilst improving care for some patients, could widen inequalities?*
- *Could the technology generate a negative value (i.e. costs would be more than gains) for some stakeholders?*
- *Are there any safety concerns about the technology or the care model it supports?*
- *Did the technology require extensive changes to organisational routines, pathways and technical infrastructure?*

1. **To what extent do you think that your unit/department/team/hospital has been ready for these technologies/innovations?**

*Prompts:*

- *Is there a good fit between the your team’s mission and the innovations?*
- *Are there any key people (especially senior management) who oppose the innovations or are unconvinced of their value?*
- *Are the business cases strong and accepted?*
- *Are the implications (e.g. work required) of introducing, implementing and evaluating the technology being adequately and realistically assessed?*
- *If money is needed, are budget lines allocated?*

1. **To what extent do you this work has been realistically assessed and adequately resourced at the organisational level ?**

*Prompts:*

- *Work to bring people on board and develop a shared vision for the change?*
- *Work to develop, implement and mainstream new care pathways and processes?*
- *Work to coordinate the project across more than one team or sector?*
- *Work to evaluate and monitor the change?*

1. **How would you rate [REDACTED]’s health service overall capacity to take on technological innovations?**

*Prompts:*

- *How strong is the leadership?*
- *Are the organisation’s mission and values clear?*
- *How good are internal relations, especially between managers and clinicians?*
- *Would you describe the management structure as flat and egalitarian or top-down and hierarchical? (For example, are individual departments discouraged from horizon-scanning for new products and ideas, and are they frowned upon if they introduce innovations?)*
- *What is the organisation’s track record of introducing any kind of change?*
- *To what extent are there slack resources (people or money) to channel into innovative projects?*
- *To what extent is it a learning organisation (in which staff are encouraged to meet and talk about new ideas and projects, there are measures in place to capture data and monitor progress, and risk-taking is encouraged?*
- *What is the current level of digital maturity?*

1. **In your opinion, how well has the development and roll-out of these decision support systems been sustained over time (if at all)?**

*Prompts:*

- *Is training ongoing?*
- *What kind of modifications have been made?*
- *How would you rate the adaptability of the technology to your context and work processes?*
- *What barriers or enablers would you identify to sustaining CDSS in your work environment?*

1. **In your opinion how well has the development and roll-out of these decision support systems aligned with organisational strategic priorities and/or outcomes?**
2. **After reflecting on your experiences working with some of the computerised systems we discussed, is there anything else you would like to add to about how they are used within the health service to improve patient care?**

***[END OF INTERVIEW]***
